# The role of health systems for health security: a scoping review revealing the need for improved conceptual and practical linkages

**DOI:** 10.1186/s12992-022-00840-6

**Published:** 2022-05-15

**Authors:** Garrett Wallace Brown, Gemma Bridge, Jessica Martini, Jimyong Um, Owain D. Williams, Luc Bertrand Tsachoua Choupe, Natalie Rhodes, Zheng Jie Marc Ho, Stella Chungong, Nirmal Kandel

**Affiliations:** 1 School of Politics and International Studies (POLIS), University of Leeds, Leeds, LS2 9JT UK; 2 Institute of Population Health Sciences, Centre for Clinical Trials & Methodology, Queen Mary University London, London, E1 2AD UK; 3 School of Public Health, Université Libre de Bruxelles, 1070 Brussels, Belgium; 4 Department of Government and International Relations, The University of Sydney, Sydney, Australia; 5 School of Politics and International Studies (POLIS), University of Leeds, Leeds, LS2 9JT UK; 6 World Health Organisation, WHO Health Emergencies Program, 1211 Geneva, Switzerland

**Keywords:** Health systems, Health security, Health workforce, COVID-19, Rapid review

## Abstract

**Background:**

Practical links between health systems and health security are historically prevalent, but the conceptual links between these fields remain under explored, with little on health system strengthening. The need to address this gap gains relevance in light of the COVID-19 pandemic as it demonstrated a crucial relationship between health system capacities and effective health security response. Acknowledging the importance of developing stronger and more resilient health systems globally for health emergency preparedness, the WHO developed a Health Systems for Health Security framework that aims to promote a common understanding of what health systems for health security entails whilst identifying key capacities required.

**Methods/ results:**

To further explore and analyse the conceptual and practical links between health systems and health security within the peer reviewed literature, a rapid scoping review was carried out to provide an overview of the type, extent and quantity of research available. Studies were included if they had been peer-reviewed and were published in English (seven databases 2000 to 2020). 343 articles were identified, of those 204 discussed health systems and health security (high and medium relevance), 101 discussed just health systems and 47 discussed only health security (low relevance). Within the high and medium relevance articles, several concepts emerged, including the prioritization of health security over health systems, the tendency to treat health security as exceptionalism focusing on acute health emergencies, and a conceptualisation of security as ‘state security’ not ‘human security’ or population health.

**Conclusion:**

Examples of literature exploring links between health systems and health security are provided. We also present recommendations for further research, offering several investments and/or programmes that could reliably lead to maximal gains from both a health system and a health security perspective, and why these should be explored further. This paper could help researchers and funders when deciding upon the scope, nature and design of future research in this area. Additionally, the paper legitimises the necessity of the Health Systems for Health Security framework, with the findings of this paper providing useful insights and evidentiary examples for effective implementation of the framework.

## Key questions:

What is already known?

- Conceptual and practical links between health systems and health security are under-studied
- The need to address this gap gains relevance in light of the COVID-19 pandemic

What are the new findings?

- A majority of existing research prioritizes health security over health systems
- Health security is treated in terms of exceptionalism focusing on acute emergencies
- Health security is often seen as ‘state security’ not ‘human security’

What do the new findings imply?

- There is a promising, yet underdeveloped, research base demonstrating how specific improvements / investments in health systems and health system building blocks can enhance long-term health security

## Introduction

According to the World Health Organization (WHO), health security relates to ‘*the activities required, both proactive and reactive, to minimize vulnerability to acute public health events that endanger the collective health of populations living across geographical regions and international boundaries’* [1]*.* Health systems are defined as the six components of the WHO Building Blocks, comprising all the resources, organizations and institutions that are devoted to producing interdependent actions aimed principally at improving, maintaining or restoring health. This definition also includes the WHO common goods for health framework as a means for delivering on the Building Blocks.

Academically, the field of health systems has focussed on non-communicable diseases (NCDs) due to their impact on day-to-day health system capacities, whilst health security has focussed on communicable diseases due to the potential for such diseases to spread. However, there is an important relationship between health security and health systems, and conceptual and practical links between notions of public health and security are historically prevalent [2, 3]. These links refer to the factors that are either common to both health systems and health security, or those that relate to health systems and its impact on health security, or vice versa. For instance, if health systems can reduce NCDs, then they can also reduce comorbidities and susceptibility to infectious disease outbreaks. Further, if a health system has processes in place to address NCDs, then such a system will be better able to act as a first line of defence during a communicable disease outbreak. Related, if a health system does not have an adequately trained health workforce, then the health system will be unable to prepare for or respond to crises that occur, such as pandemics, which would pose a threat to health security.

Another example of the links that exist between health systems and health security emerged from a brief survey of disease control literature. This search illustrates a longstanding concern with emerging disease risk and its security threats to governments [4, 5], economies [6] social cohesion [7], and general population security [8]. In addition, at least rhetorically, the relationship between strengthened health systems and enhanced health security is present within the public health lexicon. Links between building ‘strengthened’ or ‘resilient’ health systems for the promotion of health security is implied or roughly outlined in many global health frameworks [1], including being grounded in the International Health Regulations (IHR) [9] as well as in discussions within key global institutions (especially after Ebola), such as the WHO, the Governments of Seven (G7) and Twenty (G20), the World Bank, and the United Nations Security Council [10–13]. Moreover, nascent links between health system strengthening and health security have started to emerge in several national strategic plans and in global initiatives such as the Global Health Security Agenda (GHSA) and One Health, which aim, inter alia, to better facilitate the implementation of the IHR [9].

However, previous rhetoric surrounding the need to strengthen health systems for health security has not produced commensurate investments and has noticeably tailed off after many of the epidemic events of the last two decades. This gap gains relevance in light of the COVID-19 pandemic since the variability of disease burden and response has clearly demonstrated a crucial relationship between health system capacities and effective health security response. In most countries, across high, middle and low-income settings, health system deficiencies hampered effective response [14–20]. These included a lack of emergency planning and leadership [14], poor health communication combined with limited population wide health literacy [15], equipment shortages [16, 17], disruptions to medical supply chains [18], and incapacities to handle even limited increases in caseload frequency [19]. Other deficiencies have included poor information on health systems capacities, and the tenuous assimilation of private sector providers within national systems, this having led to often chaotic and ad hoc engagement with governments in national pandemic responses [20]. As such, what the COVID-19 pandemic has underscored is that there can be no genuine and resilient global health security without strong health systems characterised by essential capacities and resources present at all levels.

Acknowledging the importance of developing more resilient health systems globally, and the need to better elucidate the linkages between health security and health systems, the WHO developed the Health Systems for Health Security framework [21], published in 2021. Researchers at the University of Leeds acted as key collaborators in its development by providing evidence and advising on the development of the framework as part of a long-standing collaboration with the WHO. The framework aims to help countries, partners and the WHO be better prepared to manage public health events by closing the gaps in health systems that can lead to improved preparedness. It lays out components of health systems for health security, highlighting the foundational elements of health systems and other sectors, aiming to: a) Foster a common understanding of what health systems for health security entails and how it contributes to better emergency preparedness to prevent, detect and respond to threats and events, and; b) Identify capacities that are required for health security, including capacities from the revised WHO benchmarks for the International Health Regulations (IHR), new health system security benchmarks, a new dynamic preparedness matrix, as well as the identification of investment capacities required in other related sectors.

The framework describes health systems in terms of the WHO health systems building block model [22] and their links to the WHO benchmarks for IHR capacities [23]. The building blocks are used as a heuristic for the framework as they define interlocking essential health system components that all systems (regardless of how they are organized) have to undertake, to manage, promote and deliver effective national health. These building blocks are intertwined and thus, approaches should take into consideration the whole of the system rather than single building blocks or partial subsets. The building blocks are: 1) adequate leadership and governance, 2) a well performing health workforce, 3) a good health financing system, 4) responsive health services, 5) a well-functioning health information system, and 6) access to essential medical products, vaccines and technologies [22, 24]

The Health Systems for Health Security framework offers a valuable approach in forging links between health system strengthening and health security in a practical manner. However, it is important to illustrate and establish linkages between health systems and health security with supporting evidence. What our study identified is that there is limited readymade evidence that comprehensively and explicitly synthesises research on health systems, health systems strengthening and its positive impacts on health security. Although there is a wider research body connecting the IHRs to various notions of population health and global health security, and there is research making links between health system preparedness and health security, there is limited evidence and research that explicitly examines health security by means of strengthening the WHO health system building blocks. In addition, there is seemingly no research that examines all of the six building block components from a health security perspective. As a result, it is necessary to explore and to preliminarily assess the potential size and scope of available research literature and evidence in support of Health Systems for Health Security.

Therefore, as part of the University of Leeds collaboration with the WHO on the Health Systems for Health Security programme of work, a rapid scoping review was carried out. The purpose of the review was to explore how health system thinking, in the form of the six building blocks, has been used and how it can be used to make conceptual and practical links between health systems and health security within the peer reviewed literature. The review was guided by the following three research questions: 1) What is the existing peer reviewed evidence linking health systems to health security?; 2) What are the essential elements and characteristics of a ‘strengthened’ health system for health security as understood through the heuristic of the WHO Building Blocks?, and; 3) What examples exist to help demonstrate how improvements in health system core components can improve health security?

## Methods

A systematic search was conducted to identify literature on the relationship between health systems and health security. Given the lack of synthesised evidentiary material related to the association between health systems and health security, the complex and heterogenous nature of its agenda, and the timeframe available, a rapid scoping review was considered the most appropriate approach [25–28]. The review adopted an augmented methodology as developed by Arksey and O’Malley [29] and further refined by Levac et al. [30] and the Joanna Briggs Institute [31]. The aim of the review was to help the WHO 1) locate and clarify key conceptual and practical linkages between health systems and health security in the peer reviewed academic literature; 2) identify key characteristics or factors related to these linkages; 3) act as a precursor to a systematic review, and; 4) identify and analyse knowledge gaps.

### Search strategy and selection criteria

**Fig. 1:**
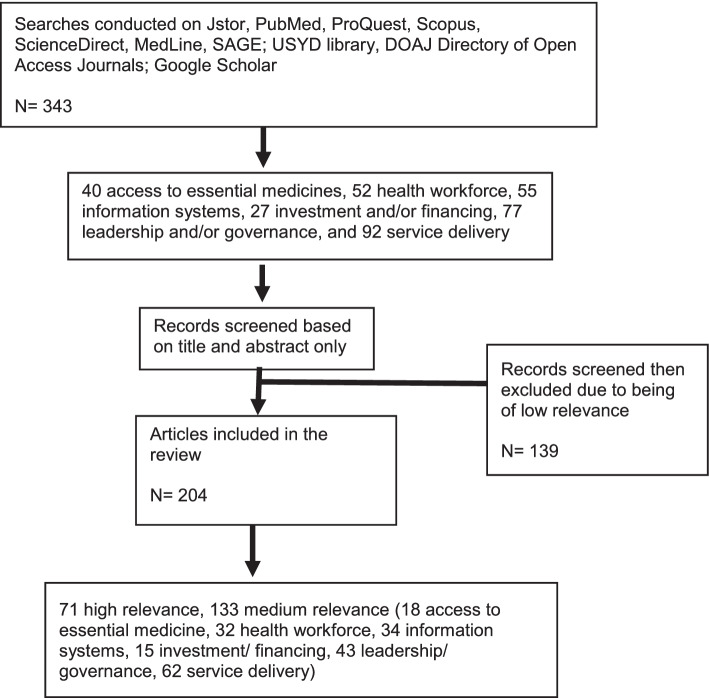
Flow diagram of the scoping review process

Seven electronic databases: Jstor, PubMed, ProQuest, Scopus, ScienceDirect, MedLine, SAGE, two institutional library repositories: USYD library, DOAJ Directory of Open Access Journals, and one search engine: Google Scholar were searched. The platforms were chosen to represent a balance between medical and social science research databases, since the partners agreed that COVID-19 exposed the need for greater interdisciplinarity in how we think about health emergency prevention, preparedness, and response. Given that the scoping review is attached to an existing policy framework, a number of key search terms pre-existed. These include resilience, emergency preparedness, WHO benchmarks for IHR capacities, health emergency and disaster risk management, disaster risk reduction, disaster management, common goods for health, and disease control. Variants and combinations of these search terms were used to identify literature. All databases were searched between August 22 and September 22, 2020 for entries between Jan 1, 1999, to July 1, 2020. This would cover the past 20 years of available literature (see Fig. 1).

Publication format was limited to peer-reviewed journal articles (as a filter for quality resulting from the peer-review process), including all types of review publications (narrative, systematic, and meta-analysis). Previous reviews were included since the aim of the scoping review was to identify key themes and trends rather than extracting data for meta-analysis (the task of a future systematic review). The publications included were all in English but were published in any country. Publications were deemed eligible for inclusion if:

- They discussed both health systems and health security, their related policies (IHR, IHR benchmarks, Health System building blocks, Common Goods of Health) and associated policy concepts (resilience, emergency preparedness, disease control, risk management and health emergencies).
- They focussed on the national health system in low, middle and/or high-income countries. Publications were also included if they focussed on national sectors outside of health systems when it could be demonstrated that a core function of the health system could not assure health security without direct assistance or input from the related sector.

### Publication selection

Search results were imported into Excel. Title and abstract screening was done by three researchers (GB, JM, JU) and was reviewed independently by a fourth researcher (GWB). The full text of the publications that were of high and/or medium relevance to the research questions were reviewed independently by three researchers (GB, JM, JU, GWB). If there was any uncertainty whether publications met the inclusion criteria, this was resolved through discussion among the four researchers. Relevance was assessed based on the following criteria:

- High: The publication makes direct links between health systems and health security (primary RQ) and provides lessons on elements and characteristics of a ‘strengthened’ health system for health security (sub-question A) with the presentation of case examples (sub-question B).
- Medium: The publication presents links between health systems for health security (primary RQ) but provides a more general reflection with few concrete lessons (sub-question A) and no case studies / examples (sub-question B).
- Low: The publication presents few if any links between health systems for health security (primary RQ).

All members of the collaboration were involved in summarizing the results and in the discussion analysis. Analysis was conducted thematically, with themes being allowed to emerge inductively based on the review findings.

### Patient and public involvement

Our study does not involve the participation of patients or any members of the public and has been approved by the WHO for publication. All literature included in this study are available from the literature databases mentioned in text.

## Results

As summarised in Fig. 1, the search yielded 343 articles across the six WHO building blocks, of those, 92 related to service delivery, 77 to leadership and/or governance, 55 to information systems, 52 to the health workforce, 40 related to access to essential medicines and 27 to investment and/or financing. 

**Fig. 2:**
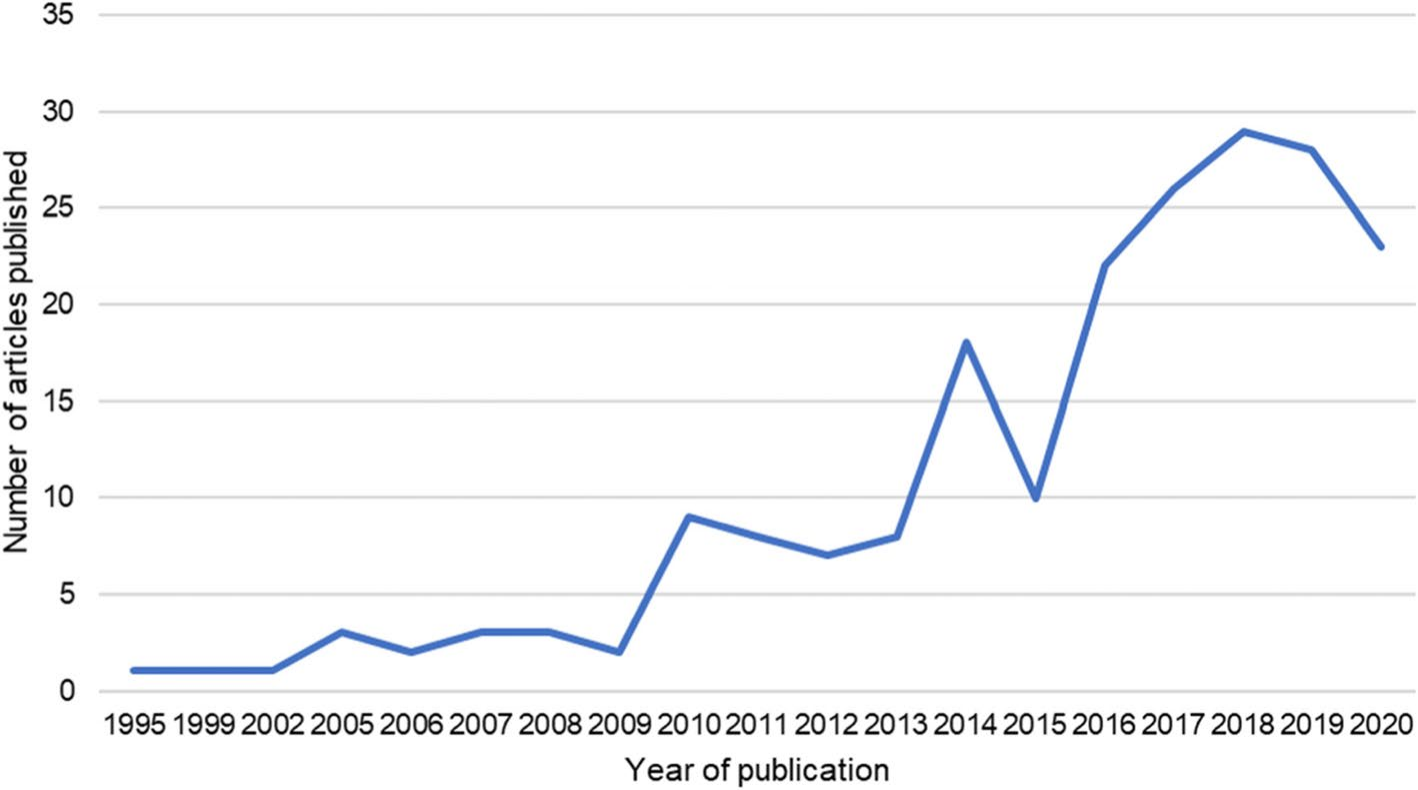
Count of publications per year on health systems and health security

**Fig. 3:**
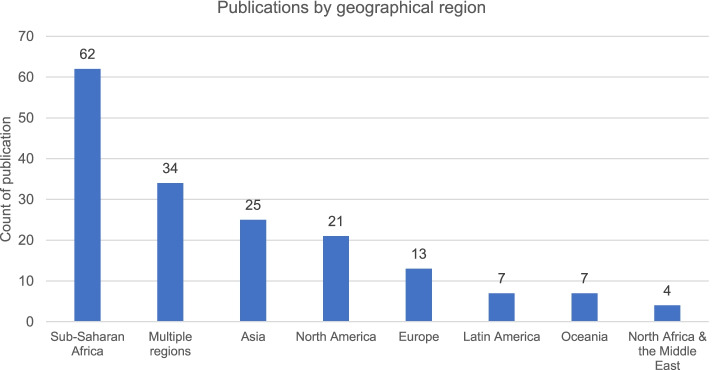
Total aggregate number of publications by geographical focus

Two hundred four were selected as being of high or medium relevance to health systems or health security and the links between the two concepts. Of those, there were 32 that covered the health workforce, and the 34 articles that covered information systems, the majority (*n* = 25 [78%] and *n* = 22 [65%] respectively) were published after 2016 (see Fig. 2). Of those published on the health workforce, most were review articles (*n* = 13, [40%]) or editorials (*n* = 8, [25%]). There were 15 articles published on investment and financing, the earliest published in 2013. The most numerous publications were published on the topics of leadership and governance (*n* = 43) and service delivery (*n* = 62). The geographic focus of the articles was largely on sub-Saharan Africa (*n* = 62), with 34 considering a range of locations (see Fig. 3).

The results are presented in two parts. The first part describes the general implications of the findings, highlighting how in much of the existing literature health security is prioritised over health system strengthening, how health security is considered in terms of exceptionalism and that there is a focus on acute emergencies rather than day to day challenges. In the first part of the results, we also highlight the framing of healthy security from a state level and describe the focus of the literature on low and middle-income-countries (LMICs). In the second part of the results, we present a series of practical examples of where the building blocks for health systems and health security have been considered in the literature and what key links can be derived from these examples. As suggested above, organizing the material in this way allowed for collaboration partners to better understand the ‘current state of play’ regarding present conceptual and practical links between health systems and health security as well as the degree to which there were existing case examples of health system strengthening for health security, which could provide useful insights and examples for the new WHO HSforHS framework.

### General implications of findings

#### The prioritization of health security over health systems

The review exposed a tendency in the literature for authors to favour a security framework over the examination of how health systems could compliment health security strategies or vice versa. The overarching role of health systems to deliver health security was considered as an aside without detailed examination in several articles, with 21.6% (*n* = 44) publications discussing mainly health systems and 8.3% (*n* = 17) discussing health security. Despite the interconnectedness of the building blocks, the review found no publications explicitly using all six WHO building blocks as a systems lens to investigate the promotion of health security.

#### The tendency to treat health security as exceptionalism

Much of the literature discussed health security in terms of being an exceptional form of response versus a concept embedded within the wider public health continuum and/or health system strengthening approach. The treatment of health security as a form of ‘exceptionalism’ meant that recommendations for improving security capacities were often isolated to a single building block, a sub-set of a building block, or ignored health systems approaches altogether, preferring to focus on the use of emergency powers [32–34]. The review did locate critiques of existing approaches to health security as anathema to a health system approach (Paul et. al. [35], highlighting how traditional health security paradigms “continue to speak in the terms of costly ‘countermeasures’ versus prevention and health system strengthening”).

Exceptionalism was also prevalent in literature discussing health security related to an individual building block or to broader system capacities. In relation to access to essential medicines, Elbe’s work on the ‘pharmaceuticalisation’ of health security was critical of how pandemics have the capacity to disrupt every aspect of society [36]. Yet, in addressing such health security crises, vaccines, and in particular their stockpiling, facilitated “a new logic of managing infectious diseases,” that seeks to implement measures to control a pathogen’s disruptive impact, rather than substantively address the cause of disease [37]. As a result, the literature implied that decision-makers pursue policies that minimise disruptions and costs, versus long-term initiatives aimed to improve overall health system capacity to prevent, prepare and respond to emerging threats [38, 39].

Exceptionalism was also reflected through a strong focus in the literature on surveillance, where a ‘strengthened’ health system was often measured against its immediate capacity to detect, alert with early warning and respond to emerging risks as opposed to measurements based on the system’s capacity for disease risk management. Njeru et. al. [40] argued that health security often creates siloed improvements mainly in public health surveillance systems, diminishing wider system benefits that could be gained via the implementation of new technology. With regards to finance and investment, most of the literature highlighted the inability of security approaches to appropriately engage with context-specific obstacles and moderators, overlooking particular system and service delivery related factors such as geographic remoteness [41], difficulties in the transfer of subsidies [42] or in the case of the West Africa Ebola outbreak, the lack of “a smooth transition from short-term humanitarian relief to longer-term development-oriented programmes”, indicating wider problems in leadership and governance [43].

#### Tendency to focus on acute health emergencies

Relatedly, the analysis of the public health topics and/or events addressed shows a clear tendency in the literature to focus on acute health emergencies and much less on preparedness (for instance, see [44, 45]). As shown in Table 1, most articles discussed emergencies such as infectious diseases (*n* = 120). This is compared to 19 discussing preparedness and 2 discussing non-communicable diseases (NCDs). Although this result is fitting with an expected division of labour between health systems research and research on health security, it does illustrate the scale of this division and thus the potential for missed interdisciplinary insights when combined.

**Table 1:** Number of publications addressing a specific public health topic or event

Public health topic/ event	Number of publications
Infectious diseases	116
Biological and chemical threats including terrorism	9
Natural hazards	9
Maternal and child health	5
Non-communicable diseases (NCDs)	4
Antimicrobial resistance (AMR)	3

Among the infectious diseases addressed, the 2014–2016 Ebola outbreak in West Africa was the most studied (*n* = 35). Ebola was often studied and discussed to reflect on particular weaknesses of health systems in low-income countries (LIC), but also, as a heuristic to analyse the failures related to global health governance and its inability to effectively respond to Ebola. These articles made general arguments for the need to support and strengthen fragile health systems as well as to enhance global mechanisms to respond to public health events of international concern [46]. Where more detailed health system recommendations were discussed to enhance health security, they tended to focus on one or two subsets of a WHO building block(s). In terms of service delivery, a major theme related to information systems during acute emergencies via the delivery of surveillance services, which did not cover general issues related to the delivery of healthcare services during the emergency. In relation to emergency capacities, a majority of the articles focused on appropriate information infrastructure, targeting regulations and capacities needed to collect, transfer and analyse data effectively. Information functions were considered to be critical for public health preparedness [47], namely for the prompt detection and containment of epidemic-prone diseases. Only a few articles dealt with “routine” health system capacities, such as those related to NCDs (*n* = 2), instead mainly focusing on issues related to chronic stress [48], and characteristics of health systems, such as trust [49], or quality of services [50]. It is worth noting that the few articles dealing with routine system capacities as a necessary baseline for general health security were unusual in that they adopted a wider perspective. These considered health security as part of an overall resilient health system, where investments in Common Goods for Health, or efforts to strengthen health systems towards Universal Health Coverage (UHC), were argued to produce long-term health security dividends (for instance see [51, 52].

#### Conceptualisation of security as ‘state security’

The conceptualisation of health security in terms of state security, and not of individual health or population security, was another emergent theme. Broader ambiguities existed within the health security literature where the meaning of the two key terms, health and security, “varied depending on the immediate pathogen posing a threat, reflecting the dynamism of this concept” [53]. Indeed, the notion of security did not lend itself to a consistent definition, constituting different things for different audiences and authors. Nevertheless, there was a tendency within the literature to treat health security risks as being a threat to national interests, which often implied population health, while highlighting issues of diminished economic competition, effect on military readiness, governmental control, and civil unrest [54].

Notions of national health security featured particularly in publications on leadership and governance, especially around notions of maintaining political control or national power [55, 56]. The decision in some countries not to share H5N1 samples notes how such decisions may be based on ‘*the preservation of national sovereignty*” [38]. This same topic was covered by Kamradt-Scott and Lee in their discussion of the 2011 Pandemic Influenza Preparedness Framework [39]. The prioritisation of national interests demonstrated “competing discourses, domestic and international, which emphasize pre-existing political priorities and serve to de-emphasize disease control strategies which break existing norms”, leading to a situation where the securitisation of certain diseases had to reflect “local political, economic and social factors for its character and success” rather than merely reflect epidemiological evidence and public health priorities [55]. There is, as a result, a strong national security characteristic within the literature analysed, which received consistent critical response, a trend seen throughout all six building blocks, yet coalescing largely around three policy areas of surveillance, pharmaceutical stockpiling, and bioterrorism [37, 57]. 

#### Focus on ‘the south’ and LIC/LMICs as the source of the global health security ‘problem’

**Fig. 4:**
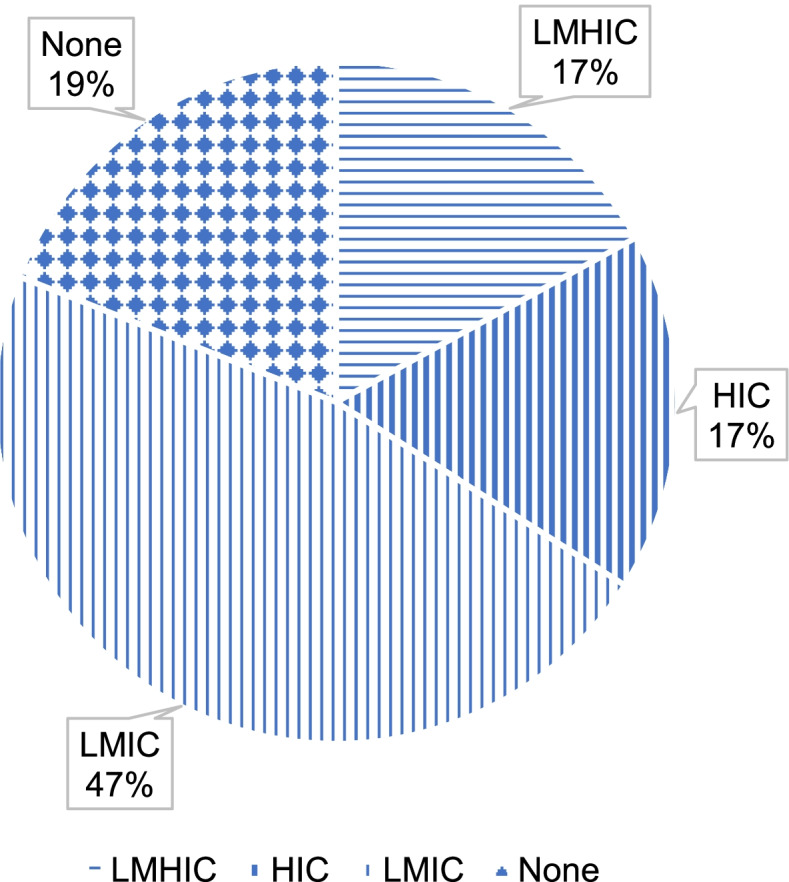
Number and proportion of publications (*n* = 203) by country income group. LMIC = low- and middle-income countries; HIC = high-income countries, LMHIC = low- and middle- and high-income countries

Geographical coverage of the literature shows a clear tendency to focus on low-and middle-income countries (LMIC), and on Sub-Saharan Africa in particular. Almost half of the publications (*n* = 93) referred specifically to LMICs, while only 17% were related to high income countries (HICs) (*n* = 35). Twenty-nine publications (14%) adopted a wider comparative perspective including data and examples from countries with different income levels. The remaining 23% of the publications had no geographical focus but proposed a more general reflection either on global health governance or on specific conceptual topics based on literature reviews (*n* = 47) (See Fig. 4). Among the publications which had an explicit geographical target (*n* = 130), initiatives and events occurred in Sub-Saharan Africa were the most covered (*n* = 58). With respect to the geographical coverage, it is also worth noting that of the articles specific to North America (*n* = 21), 13 discussed Mexico.

This focus on LMIC and Sub-Saharan Africa reveals the tendency to consider ‘the Global South’ as the potential source of global health security issues. The situation in countries like Afghanistan or Uganda [58, 59] which are prone to epidemics and where capacities to detect and respond to diseases outbreaks remain limited, is described as hampering global health security broadly. The attention given to the 2014–2016 Ebola outbreak in West Africa is also a case in point. According to McPake and colleagues, this event confirmed “how the world's weakest health systems threaten global health security” [60, 61]. The articles related to high income countries (HICs) confirm this concern about the risk of diseases from the Global South spreading globally. Out of the 34 articles referring to Europe and North America, 57% (*n* = 18) dealt with the capacity to respond to pandemics [54, 54, 62–65], and infectious diseases like Ebola or Zika [66–73], or tuberculosis [67], with travel medicine services [74], as well as with actions that are/should be taken internationally to strengthen global health security [57, 75].

#### Under-representation of NCDs

The focus on acute health emergencies results in the under-representation of non-communicable diseases (NCDs) among the specific health topics addressed in the scoping literature. This is excepted given that research in health security has traditionally been on communicable disease control measures and policy. Yet, as discussed below, this represents an area where cross-disciplinary research would expose and respond to broader health threats in general and the relationship between communicable diseases, like SARs-CoV-2, and multimorbidity underwritten by NCDs. As Table 1 shows, only 2 articles specifically addressed NCDs, either in general as a group of diseases [76] or in relation to cancer [77] and cardiovascular diseases [78]. This represents just 1% of all articles included in the review. The articles acknowledged the disparity between the high prevalence of NCDs as the leading causes of mortality and the little investment and attention given to these diseases. In particular, Reis and Cipolla [78] stress the imbalance existing between the priority given to epidemics, bioterrorism and climate change by international health security initiatives and the fact that today the leading causes of mortality worldwide are non-infectious, inviting authorities to address broader determinants of health.

### Practical examples of strengthening the building blocks to achieve greater health security

A key component of the collaboration was to locate practical examples of how health system investments across the six WHO Building Blocks resulted in co-benefit health security improvements. A number of articles offered specific case studies of how strengthening building block capacities resulted in better system readiness, resilience and security. Twelve case studies (two per building block) were included in an Appendix within the WHO Health System for Health Security framework. Indicative examples of case studies are discussed below [21].

#### Adequate leadership and governance

Indonesia was noted for improving emergency preparedness and IHR 2005 compliance via broader health system investments [79]. Since the Avian Influenza A(H5N1) outbreaks in 2005, Indonesia established a series of plans, guidelines, and committees to control avian influenza and prepare for future pandemics (culminating in the *National action plan for health security 2020–2024)*. This plan integrated Indonesia’s *National medium-term development plan 2020–2024* to include a focus on health system strengthening based on primary health care. At local levels governance and leadership initiatives were also highlighted as playing a crucial role in how Mexico City and New York City responded to Influenza AH1N1 in 2009 [62]. In both cases, pre-existing emergency plans were specifically designed to facilitate intersectoral linkages and decision-making alongside enhanced surveillance protocols and training. As a result, the literature often stressed that the development of strategic policy frameworks and effective oversight of their implementation are among the key functions of health security governance. In particular, the importance of pre-crisis emergency or pandemic plans was mentioned [80], as well as the need to develop plans for post-event recovery [81]. In several cases, the impact of having already experienced health emergencies was noted as a key driver of change underpinning countries’ preparedness [82]. Key articles in this area referred to the capacity of governments to rapidly coordinate activities, to promote the engagement of multiple stakeholders, including civil society, the private sector, and international players, as well as to learn from experience with adaptive strategies [83, 84].

#### A good health financing system

In terms of system investment and security promotion, a study of Thailand examined how health system and universal health coverage policies were strategically combined as part of a larger national health security agenda [85]. Thailand’s Universal Health Coverage Scheme (2001) and the Health Security Act (2002) invested in local health system strengthening as a requisite infrastructure for universal health coverage as well as more sustainable and cost-effective health security measures. These reforms were achieved through the political elevation of health as a means through which broader national development and security could be achieved, demonstrating investments towards universal health coverage will have positive ripple-effects across all sectors in the promotion of long-term national interests [85–87].

Similar links between strengthening investments in health system building blocks, universal health coverage and health security were often reported in response to the Ebola outbreak, particularly in West African post-Ebola contexts [60]. Again, links between the delivery of universal health coverage and long-term security prevention and preparedness were discussed as essential, with particular emphasis on healthcare workforces. However, it was highlighted that an important component of delivering on these commitments is reliable financing and a steady growth in health budgets, where initial reluctance to these investments need to be framed as providing future cost-savings and recognition of the long-time-horizons for population health outcomes [51].

A focus on enhancing health system ‘resilience’ was often noted to mitigate service delivery shocks associated with acute health emergencies. A study in Lebanon noted that the country was able to maintain the continuity of services both for citizens and refugees during the Syrian refugee crisis (2011 to 2013) thanks to previous reforms to reduce out-of-pocket expenditures and ensure uninterrupted financial coverage, as well as financial commitments to increase the number of primary health centres in the national network [88]. A study by Memish et al. [89] revealed how interventions made by Saudi Arabia have helped public officials to prevent and mitigate the outbreak of infectious diseases during the Hajj. These interventions included increased attention to vaccination programmes and travel medicine protocols, free medical care to pilgrims in hospitals (including for critical care), education campaigns, safe water and food, and increased diseases monitoring as well as surveillance at points of entry. These interlinking and multisectoral improvements in services resulted in no occurrence of a major outbreak at the Hajj over the past decade, despite the emergence of several new coronavirus and influenza viruses.

#### A well-functioning health information system

A study looking at information system improvements in Uganda after the Ebola outbreak noted that investments in community-based surveillance systems were important to ensure country preparedness and health security and should be viewed as foundational to the health system and not just an exceptional measure used during health emergencies [90]. Furthermore, a literature review on health system strengthening showed that building novel surveillance systems can improve both clinical care and health system preparedness for health threats [91]. Beyond this, the review findings also provide evidence that small to medium health information improvements can significantly underwrite health security, such as more fully integrating national laboratory systems [92, 93]. In the Democratic Republic of Congo, low-tech improvements in data management and training resulted in more rapid and effective Ebola response [47, 94], while in Cyprus, streamlining information sharing and the use of information technologies showed evidence of improved emergency preparedness as well as improved delivery of routine services [33]. Evidence also supports the health security benefits of building better networks and shared learnings between national and regional laboratory and information systems, in both LMIC [91, 95–99] and HIC settings [100].

#### A well performing health workforce

The need to strengthen workforce epidemiology capacities to collect and analyse data was a consistent theme [58]. Several articles examined country level initiatives like the Field Epidemiology Training Programs (FETP) or the African Field Epidemiology Network (AFENET), both of which have received the support of the US Center for Diseases control [58, 99, 101]. More system-wide and multisectoral reflections on how to adapt applied epidemiology training to current challenges (e.g. technological innovation, multidisciplinary teams, delivery of population health services, and global health security) was also present [102]. The review revealed considerable attention to the laboratory workforce capacities as the key basis for the promotion of health security [57], particularly a focus on the capacity to detect infectious diseases like Ebola and Zika (for instance see [72, 103]). Initiatives to support capacity building of workforce at this level were also reported, with international initiatives, such as the International Laboratory Capacity Building Program established by the American society for microbiology [104] highlighted as promoting health security. Beyond this, local initiatives like Uganda’s program to enhance the capacity of the health workforce were also linked to the promotion of health security. As an example, Tanzania’s training program targeting both epidemiology and laboratory capacities were understood to reflect a positive knock-on effect of health security rationales [105]. It should be noted that a focus on wider workforce capacity needs and system level improvements were completely absent.

#### Access to essential medical products, vaccines and technologies

Technologies were highlighted as being important to the identification of pathogens. Borchert and colleagues [106] highlighted how Uganda’s development of cold-chain systems for specimen transport, improved test algorithms for priority pathogens, and efforts to standardize distribution operating procedures are key health security commitments. Related to access to essential medicines, the example of Indonesia again was noted in the literature as representing innovative linkages between health security and health system development [107], where affordable access to essential medicines was a priority within its National Health Insurance System, providing lessons as the world’s largest single-payer scheme.

## Discussion

### An undervalued link between health systems and health security

A key observation from the scoping review is that explicit linkages between health system research and health security were largely absent in the literature. Even when specifically targeting literature of ‘high relevance’ via our inclusion criteria (*n* = 63, [30.8%]), rarely were the terms conceptually or explicitly linked in any substantive way, reflecting a tendency in the literature to treat them largely as separate fields of study or to treat linkages as self-evident or implicit. Although it is understandable that some degree of subject specialism and a division of labour must exist, this observation nevertheless generates a number of important questions and implications about how we currently understand the relationship between health systems and health security.

First, the tendency to treat health systems and health security as distinct fields of study runs counter to more recent calls for the integration of securitised issues within existing health systems [51] and fails to reflect nascent policy discourses attempting to champion long-term health system improvements as a form of promoting health security [108]. The WHO has also been working on a position paper on building resilient health systems for universal health coverage and health security [109].

One explanation for the historical lack of linkage is that the respective research agendas merely maintain fundamentally different approaches to health. The review found that health security is often understood as an exceptional condition or that it relates primarily to acute health emergencies, thus elevating health security as something that rests outside the routines and discussions of normal health policy [110]. This understanding of health security as a ‘state of exception’, framed too by the broader contexts as described in previous reports [1], could explain why much of the literature reviewed focused on acute infectious disease or biohazards. This also may explain inadequate COVID-19 readiness and response, particularly in HICs, where notions of health security took the form of a ‘once in a century’ exceptionalism, with the assumption that threats would mostly affect the Global South and that HICs capacities were sufficiently prepared, creating a condition of complacency [111].

Second, the current tendency to separate health system research as distinct from the analysis of core health security competencies may help explain why global health policy has traditionally remained overly reactive and disease-specific, losing political focus and commitment once security threats are perceived as resolved. As noted in the critical literature, there is a history of health ‘securitizations’, where a particular disease threat gains rapid prominence and coverage, only to be replaced by the next infectious disease risk – e.g., the securitisation of HIV/AIDS, pandemic influenza [39] the zika virus [112], and Ebola [113]. One policy implication of ‘security’ exceptionalism and disease-specific ‘securitization’ is that it can draw attention away from the important role of primary care and other ‘mundane’ attributes of health system performance in the provision of a strengthened health system [43]. In reality, universal health coverage and health security are complementary goals. Investing in the foundations of health systems, with a focus on primary health care, improves overall health emergency preparedness in countries. This has to be done alongside all-hazards risk management and adopting a whole-of-government approach for community engagement and whole-of-society involvement [109].

Relatedly, the lack of connection between health system improvement and increased health security can also be attributed to shortcomings in the literature to substantively express their mutual inclusivity and co-dependency. This continued disconnect is surprising given that previous governance failures raised questions about the sustainability of disease-specific responses and their impact on health and development outcomes [114]. In fact, there is literature suggesting the general recognition of historically poor HIV/AIDS response, which caused “a rhetorical shift away from the silo-based approaches to development aid… calling for ‘investing in (other areas of) development for health” [115]. Similar sentiments were iterated in the aftermath of the West African Ebola epidemic, where there were calls for renewed commitments to international public health arrangements and commitments, namely, via the implementation of the IHR [116]. It is this post-Ebola “wake up call” where the substantial beginnings of a health systems-health security nexus emerge [11], with the idea that, “the most effective systems are those that are in use every day and can be scaled up in an emergency” [43]. Yet, in spite of this emerging literature, our review demonstrates that there remains a key gap to the extent that health systems and health security are largely treated as distinct areas of research, with health security often separated from overarching health and development discourses [117].

Third, the siloed way health systems and health security have been approached poses several challenges for national and global health governance and our thinking about financial investment. Central to these challenges is the difficulty of presenting a holistic long-term strategy that moves beyond ad hoc reactive agendas [35]. Indeed, the need to take a broader systems approach toward longer-term health security investment is made more urgent when considering the evolving costs and risks of COVID-19 and the failures in national and global health governance preparedness and response. In terms of risks, as of July 2020, COVID-19 had already cost $10 trillion in lost GDP [118] with more expected due to lost earnings [119]. It had also cost $19.5 trillion in stimulus packages [120], and as of July 2021, has resulted in over 4,154,660 deaths globally [121]. Health systems saw the importance of being able to surge to meet the demands of such an emergency, flexibly deploy resources to areas of greatest need, and reduce disruptions to essential services. Needs of countries are different to mobilize resources to effectively respond to health emergencies in addition to their routine demands for health services. For instance, low-and-middle income countries (LMICs) may need support to quickly scale-up skilled and specialized workforce during emergencies. On the other hand, in high-income countries (HICs) with advanced health systems, the main challenge is a capacity to manage a huge surge in demand for health services in a short period of time during emergencies like in the COVID-19 pandemic [109]. Lastly, almost all countries, irrespective of income, faced challenges in the procurement of medical logistics to meet the demand for such supplies during the pandemic [109]. In terms of efficiency, wider health system investments necessary to help countries fully meet their IHR capacities would have represented a fraction of this cost, while potentially helping to deliver better public health outcomes.

### An overly state-centric understanding of health security

The review found that health security is generally conceptualised in state-centric terms, which often side-line or ignore issues of human security and wider population health challenges such as those associated with mental health or the disruption of critical health services, as the defining goal of health security [34, 122]. The dangers of employing such a security discourse lies in its implicit creation of a threat logic, where the emphasis upon risk and danger to legitimise exceptional measures is removed from altruistic motives, “locating it instead within a state-centric framework” [110]. What results is a particular type of policy, where a focus upon security and control creates unintended effects, such as the emergence of a surveillance policy bias [123], the framing of health as an issue of national integrity and border protection [13, 124]which have been argued to “prioritize western states” [125]. In relation to COVID-19, this underwriting logic helps to explain current issues of “vaccine nationalism”, where a small number of states have pre-purchased existing and future vaccine stocks (80% of projected supplies) under the rationale of promoting “national security interest’ [126]. This logic, intentional or not, has also arguably produced a number of COVID-19 related access and system inequities, where wealthier countries have been slow to fund ACT-A efforts (estimated $38 billion funding gap), and when they do finance, have favoured the ACT-A Vaccine Pillar with little given for the ACT-A Health Systems Connector [127].

What is more, much of the literature appears to assume the ‘Global South’ is the ‘issue area’ for global health security, while also reinforcing disjointed solutions to a global problem, especially if advocacy on investing in IHR (2005) is overlooked [128]. This has been noted to create bias, with an over emphasis on strengthening surveillance services at points of entry [129] and discriminatory migrant health policy [50]. One implication is that the lack of a more holistic approach ignores evidence on how the integration and strengthening of healthcare services in some LMIC allowed them to strengthen not only their health systems, but also broader health security [88, 130]. A focus on the Global South as merely a source of external threat or area of concern, instead of as also places that can offer opportunities for learning, may result in lost opportunities for improved health security, particularly in terms of prevention policies, system preparedness and health system adaptability.

Possibly as a result of the state centric view and focus on the global south as a source of health threats, there has been a failure to sufficiently link universal health coverage and health security. As recalled by Kutzin and Sparkes [109], universal health coverage and health security are intertwined, since the capacity of health systems to ensure individual health security (e.g. by providing people with equity in service use, quality of care, and financial protection) is intrinsically linked to building collective health security. Reforms undertaken in Thailand and Mexico are good examples of this joint effort towards universal health coverage and health security [85, 131]. More often however, our findings suggest that universal health coverage and health security are considered separately. Recent progress in bringing the two together has been accelerated by the pandemic, including through the publication of the WHO framework on health systems for health security. This corresponds with the findings of Erondu and colleagues [132] who suggest this as a missed opportunity, which calls for one unified complementary health system, integrating both population-level health services and emergency preparedness and response functions. Recent reports by the current review Committee on the functioning of the IHR during the COVID-19 response, previous similar committees for other health emergencies, the Global Preparedness Monitoring Board, and the Independent Panel for Pandemic Preparedness and Response have likewise alluded to these issues and made recommendations on the need to address them.

### Limitations

This review has a number of potential limitations. Although the research team discussed overall themes and findings throughout the review process, only one reviewer was involved in performing study selection and data extraction for each WHO building block and search category, which increases the risk of error and possibility of bias being introduced into the review process. Furthermore, because grey literature, studies not in English, and articles published before 1999 were excluded, there is an assumption that important results and unpublished data were missed. Since the inclusion criteria required explicit connections between health systems and health security to be present, there is high probability that many important studies demonstrating implicit links between health system improvements and their implications for health security promotion were missed. Finally, whilst rapid scoping reviews are often used to inform policy frameworks (due to their quickness with a reasonable level of methodological rigorousness and transparency), in the context of more abstract or broad topics, such as those discussed in this paper, the body of evidence can be difficult to corral effectively and there are trade-offs in terms of comprehensiveness. As mentioned earlier, the aim of this review was merely to get a general ‘lay of the land’ so as to identify existing gaps as well as where explicit conceptual and practical links between health systems and health security have been made. The aim of the follow-up systematic review will be to address these limitations.

## Conclusion

To our knowledge this is the first attempt to synthesise evidence from a broad and diverse global literature examining the linkages between health systems and health security. The study confirmed that these linkages remain significantly underdeveloped, with just over 50% of the yielded material in the present review explicitly making conceptual links or providing practical examples of health system strengthening toward health security.

As a result, health systems and health security were often treated as distinctive and separate domains, instead of being two sides of the same coin. Although core health system capacities to enhance health security were often singled out, such as laboratory capacity and surveillance, these investigations often remained siloed, diminishing complimentary interconnections to wider health system functions or goals. The findings also highlight that, in cases where connections were made, there is a tendency in the literature to prioritise security goals, or to focus on the examination of individual security elements, giving far less coverage to the examination of overall health system improvements as being a crucial part of a health security approach.

Accordingly, the relationship between health systems improvements as a means for enhanced health security, and vice versa, requires greater research attention. In particular, there exists a need to fill evidentiary gaps about how investments in health system capacity building can generate returns on better long-term health security. For example, research should seek to explore what kinds of investments and/or programmes can reliably lead to maximal gains from both a health system and a health security perspective. These should be accompanied by investments in health emergency preparedness, supported by investment cases, and the incorporation of lessons from the pandemic, such as the important role of community resilience and preparedness, increasing access to healthcare and gender in a spirit of continual improvement and building back better for the future. There also needs to be increased attention on the important role of gender in preparedness for health emergencies. Its impact on health security and health systems has been evident during this pandemic.

Although there is a recognition in the literature that health system capacity goes hand in hand with emergency preparedness, which can enhance health security, these links are often made intuitively, limitedly, or spuriously, and thus require further evidentiary support. In addition, the findings from this report may to some merely reinforce already existing intuitions about existing gaps between health system research and health security. As a result, our efforts here have been to more systematically evidence these gaps and to present them more comprehensively across a number of issue areas.

As a means to address the gaps identified, and in the wake of recent major outbreaks, including the COVID-19 pandemic, the increasing interest for "health system for health security" related-matters is an opportunity we can build on to support further research in this area. In particular, these findings confirm the legitimacy of the WHO Health Systems for Health Security framework by evidencing the need for such an approach, which was the question at the heart of the collaboration between the WHO and the University of Leeds. Moreover, linking system strengthening to long-term health security offers a potentially fruitful discourse for enhanced global health diplomacy and the financing of global common goods for health [133–135]. In response, the framework aims to bridge conceptual and practical gaps by facilitating a common understanding of what health systems for health security entails and how it contributes to better global health security. In essence, it understands health security and health system strengthening as synonymous co-beneficiaries. It offers a practical whole-of-society approach for global health security through the combination of health security capacities and components from health systems and other sectors that work in synergy to meet the demands imposed by health emergencies, including severe pandemic threats. Moreover, the framework specifically locates primary care and community level strengthening efforts as key mechanisms to enhance wider health security. Whilst the gaps identified in the review reinforce for the need for such a framework, it also highlights potential obstacles in the framework’s implementation. The evidence required to fully operationalise the framework remains nascent, thus exposing key research gaps that require additional attention which if addressed would support the effective implementation of the Health Systems for Health Security framework.

## Data Availability

All items reviewed are publicly available on-line. The datasets created and/or analysed during the current study are available from the corresponding author on reasonable request.

## Abbreviations

ACT-A: Access to COVID-19 Tools-Accelerator
AFEN: African Field Epidemiology Netwrok
AIDS: Acquired Immunodeficiency Syndrome
FETP: Field Epidemiology Training Program
G7: Government of Seven
G20: Government of Twenty
GHSA: Global Health Security Agenda
HICs: High Income Countries
HIV: Human Immunodeficiency Virus
HSforHS: Health Systems for Health Security
IHRs: International Health Regulations
LICs: Low Income Countries
LMICs: Low and Middle Income Countries
NCDs: Non-Communicable Diseases
UHC: Universal Health Coverage
WHO: World Health Organization
